# Automated recruitment and randomisation for an efficient randomised controlled trial in primary care

**DOI:** 10.1186/s13063-018-2723-3

**Published:** 2018-06-27

**Authors:** Victoria R. Cornelius, Lisa McDermott, Alice S. Forster, Mark Ashworth, Alison J. Wright, Martin C. Gulliford

**Affiliations:** 1 Department of Primary Care and Public Health Sciences, King’s College, London, UK; 2 Imperial Clinical Trials Unit, Imperial College London, 68 Wood Lane, London, W12 7RH UK; 3 Department of Behavioural Science and Health, University College, London, UK; 4 NIHR Biomedical Research Centre at Guy’s and St Thomas’ Hospital, London, UK

**Keywords:** Automated randomisation, Efficient trial design, Randomised controlled trial, Primary care, Electronic health records

## Abstract

**Background/aims:**

Use of electronic health records and information technology to deliver more efficient clinical trials is attracting the attention of research funders and researchers. We report on methodological issues and data quality for a comparison of ‘automated’ and manual (or ‘in-practice’) methods for recruitment and randomisation in a large randomised controlled trial, with individual patient allocation in primary care.

**Methods:**

We conducted a three-arm randomised controlled trial in primary care to evaluate interventions to improve the uptake of invited NHS health checks for cardiovascular risk assessment. Eligible participants were identified using a borough-wide health check management information system. An in-practice recruitment and randomisation method used at 12 general practices required the research team to complete monthly visits to each general practice. For the fully automated method, employed for six general practices, randomisation of eligible participants was performed automatically and remotely using a bespoke algorithm embedded in the health check management information system.

**Results:**

There were 8588 and 4093 participants recruited for the manual and automated methods, respectively. The in-practice method was ready for implementation 3 months sooner than the automated method and the in-practice method allowed for full control and documentation of the randomisation procedure. However the in-practice approach was labour intensive and the requirement for participant records to be stored locally resulted in the loss of data for 10 practice months. No records for participants allocated using the automated method were lost. A fixed-effects meta-analysis showed that effect estimates for the primary outcome were consistent for the two allocation methods.

**Conclusions:**

This trial demonstrated the feasibility of automated recruitment and randomisation methods into a randomised controlled trial performed in primary care. Future research should explore the application of these techniques in other clinical contexts and health care settings.

**Trial registration:**

Current Controlled Trials, ID: ISRCTN42856343. Registered on 21 March 2013

**Electronic supplementary material:**

The online version of this article (10.1186/s13063-018-2723-3) contains supplementary material, which is available to authorized users.

## Background

Randomised controlled trials are regarded as the optimal design for evaluating the effectiveness of health services and medical interventions but the costs associated with conducting trials have increased substantially for a range of reasons. Research funders, including the National Institute of Health Research (NIHR) and the Medical Research Council in the UK, are now promoting the incorporation of ‘efficiency’ into the design or conduct of publicly funded trials with the primary aim of reducing resource requirements whilst maintaining research quality. A new initiative has also been developed by the research community to develop methods for efficient trials (www.trialforge.org/) [1]. The notion of ‘efficiency’ encompasses a broad range of methodological approaches, including innovative designs, logistical planning, and novel approaches to recruitment and outcome data collection, which may be employed to reduce the level of resources required to set up and conduct a trial or to enhance the value of trial investments by enabling longer-term follow-up in usual care settings. The use of information technology and electronic health records (EHR) data to increase trial efficiency is receiving increasing attention [2]. Use of EHR may also enable trials to be conducted pragmatically in usual places of care with inclusive eligibility criteria [2, 3]. Such trials are sometimes referred to as ‘point-of-care’ trials [4, 5]. Several cluster randomised trials using EHR have now been completed [5–7], or are in progress [8, 9], but few efficient EHR trials with individual patient randomisation have been reported. Van Staa et al. [10] reported on two pilot trials conducted through the UK Clinical Practice Research Datalink, including a study of antibiotic prescribing for exacerbations of chronic obstructive pulmonary disease and a comparison of two statin drugs [10]. This report emphasised some of the difficulties encountered in conducting trials using electronic records, including questions of research governance and logistical issues for recruitment and randomisation. The Salford Lung Study [11] provided a more positive assessment for trial recruitment through EHR but this study employed a centralised randomisation service, with outcome data collected through an augmented electronic records system developed locally.

This paper reports on methodological issues in the efficient design of a large randomised controlled trial of enhanced invitation methods for the NHS Health Check programme in England [12]. The NHS Health Check programme is a national programme for cardiovascular disease (CVD) risk assessment of adults aged 40 to 74 years in England [13]. Individuals are eligible for a health check if they are registered with an English general practice, are aged 40 to 74 years, and are free from pre-existing CVD and are not treated for elevated CVD risk. Since uptake of health checks is presently considerably lower [14] than initially projected [15], we designed a trial [16], which was funded by the National Institute for Health Research (NIHR) Health Technology Assessment (HTA) programme, to evaluate the effect on health check uptake of two enhanced invitation methods. The invitation methods employed the ‘Question-behaviour Effect’ (QBE) and the offer of a financial incentive as reported elsewhere [12, 16]. During the trial we developed and implemented methods for automated recruitment and randomisation of eligible participants. The objective of this report is to describe our experience of implementing an automated recruitment and randomisation process, and to assess feasibility and methodological issues compared to a manual ‘in-practice’ method.

## Methods

We conducted a three-arm randomised controlled trial, with individual participant randomisation, incorporating both a manual (in-practice method) and a fully automated technique (‘automated method’) for recruitment and randomisation. The trial was conducted in primary care and the aim was to evaluate the effectiveness of two enhanced invitation methods to increase the uptake of invited checks for the NHS Health Check programme. The trial was commissioned by the NIHR HTA programme. Details of the trial protocol and primary results have previously been published [12, 16].

### Trial summary

The NHS Health Check programme was introduced with the aim of identifying people at increased risk of heart disease, stroke, diabetes or chronic kidney disease [13]. The intervention was based on the QBE and involved a preliminary questionnaire being sent to individuals prior to them receiving an invitation for a NHS health check. The trial design was a three-arm, superiority randomised controlled trial and can be seen in online Additional file 1: Figure S1. General practices in two London boroughs: Lambeth and Lewisham, were invited to participate in the trial. Each practice participated in the trial for a minimum of 12 months. All participants in the consented practices, who were eligible to be invited for a health check, were included in the trial. The intervention was posted with prepaid return envelope and covering letter 7 days before the standard NHS health check invitation letter and information sheet. The trial arms were: (1) Standard Invitation to NHS health check only; (2) QBE questionnaire followed by Standard Invitation and (3) QBE questionnaire and offer of a financial incentive to complete the questionnaire followed by Standard Invitation. Participants in all three trial arms received a reminder letter to attend a health check at 3 months following the initial invitation. The primary outcome was uptake of an NHS health check within 182 days (6 months) after the Standard Invitation letter. Outcome data were extracted from EHR by members of the research team using nationally specified READ codes to record completion of NHS health checks.

### Ethics

The protocol for the trial was approved by the London Bridge Research Ethics Committee on 7 March 2013 (Reference 13/LO/0197). The nature of the intervention made individual participant consent infeasible. The senior general practitioner (GP) at each participating general practice gave written informed consent to the participation of the practice population.

### Identification of eligible NHS Health Check programme individuals

Participants for NHS Heath Check programme are identified through a cross-borough call-recall system implemented by the Primary Care Shared Services team, working in association with a commercial information technology company that provides a bespoke management information system, which is used in the management of the health check programme. Invitations to the programme are issued monthly. Eligible patients are identified from general practice information systems and an initial ‘pre-notification list’ (PNL) is prepared by the commercial information technology company and sent to general practices for review to remove any participants who the general practice considers do not meet the eligibility criteria for a health check. The final list of participants eligible for invitation (‘approved PNL’) is then forwarded to primary care shared services each month and Standard Invitation letters are then sent out.

We commissioned an automated recruitment and randomisation procedure to be implemented into the Standard Invitation process though modification of the health check management information system. As there was a significant risk to the completion of the trial if not successful we developed an alternative method of recruitment and randomisation that could be implemented through in-person (in-practice) visits to general practices. The trial was delivered through the use of these two different recruitment and randomisation procedures.

### In-practice method for recruitment and randomisation

For the in-practice method of allocation, members of the research team visited each general practice monthly to access the practice-approved PNL. Participants included in the approved PNL were allocated to one of the three trial arms using a pre-prepared randomisation list. Each month, the trial statistician drew up a computer-generated randomisation list stratified by GP practice using permuted blocks of 3 using Stata command ‘ralloc’ in Stata version 12 [17]. The randomisation list was applied to the approved PNL by the trial researcher who assigned the trial arm in the existing order of the approved PNL. Practice staff responsible for preparing the approved PNL never had access to the randomisation list.

### Automated method for recruitment and randomisation

For general practices assigned to the automated method, randomisation was performed automatically using a procedure programmed into the health check management information system. Randomisation lists were generated using a bespoke algorithm embedded in the management information system, which was written by the commercial provider’s programmer. Simple randomisation stratified by GP practice was performed monthly. Participants were automatically assigned a study ID and group allocation when the cleaned PNL was electronically approved by the general practice.

**Fig. 1:**
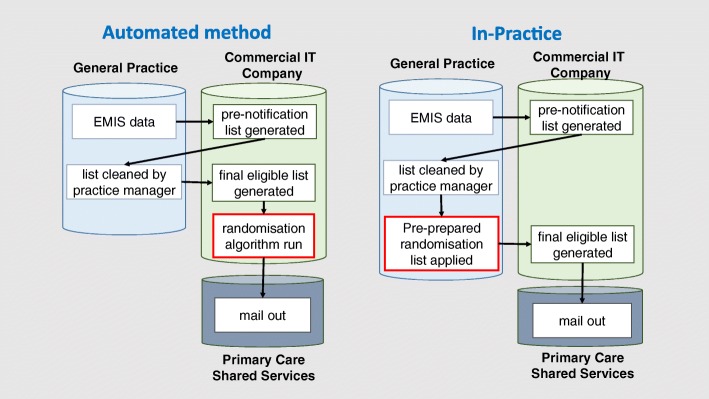
Implementation of the automated and in-practice methods for recruitment and randomisation. Implementation method for recruitment and randomisation into existing health care IT system and health checks software programme

An overview of both methods is presented in Fig. 1.

The practices were purposely selected to participate in the automated or in-practice method because we aimed to develop and test the study methods at practices that offered optimal access. The general practices assigned to the automated method represented a convenience sample. We assigned 12 general practices to the ‘in-practice’ randomisation and six to the automated method. This ratio was chosen to ensure that the trial might still be completed successfully in the event that the automated method did not succeed.

### Piloting of automated method

The automated randomisation procedure was piloted for the first 2 months to allow for amendments to be made to the randomisation code incorporated into the management information system. Data from three practices during the pilot study phase were excluded from the main trial analysis after a review of the randomisation lists revealed an imbalance in the number of participants by arm in each practice. The imbalance was the result of a programming error in the software that was subsequently corrected.

### Sample size

The trial planned to recruit 12,789 participants in order to have 90% power to detect a difference of 4% between any of the three treatment arms assuming uptake of 50% using 5% significance level adjusted for three comparisons. No power calculation for the comparison of the in-practice and automated method was undertaken.

### Statistical analysis

The planned analysis for the main trial have been reported elsewhere [12, 16]. Practice characteristics were summarised by recruitment and randomisation method. As the study was not powered to detect a statistically significant interaction between treatment arm and randomisation method, a meta-analysis was used to informally examine the impact of randomisation method for each of the three trial arm comparisons in turn. A forest plot was used to visualise the intervention estimates for the difference in uptake between arms at practice level. Heterogeneity in estimates was assessed using the I^2^ statistic [18]. In the absence of heterogeneity, estimates were combined by use of a fixed-effects model using the method of Mantel and Haenszel. Forest plots were constructed using the ‘forestplot’ package in the R programme [19].

## Results

There were 18 general practices recruited into the trial. Of these, 12 general practices were selected for the in-practice recruitment method and six were selected for the automated recruitment method, equally divided between the two London boroughs. A 2:1 ratio was implemented to minimise risk to the trial due to the unknown difficulties in implementing the automated procedure. There were 12,681 participants recruited, including 8588 through the in-practice recruitment method and 4093 through the automated recruitment method. Twelve thousand four hundred and fifty-nine participants were subsequently included in the trial analysis after excluding those included in the pilot period of automated randomisation and 38 duplicates.

Table 1 compares the practice characteristics between the automated and the in-practice method. Practices included in the automated method had slightly higher list sizes and deprivation scores but a lower proportion of ethnic minorities.

**Table 1:** Characteristics of trial practices and non-trial practices in the two boroughs in 2014–2015

	Non-trial practices	Trial general practices	
		In-practice recruitment	Automated recruitment
Lambeth	39	6	3
Lewisham	32	6	3
List size 2014–2015	6554 (4851 to 9348)	8093 (6179 to 12,568)	11,269 (7115 to 14,404)
IMD2010 score	Lewisham 31.0 Lambeth 31.2	30.2 (23.8 to 35.1)	34.6 (30.7 to 39.5)
Ethnic minorities (%)	Lewisham 46.4 Lambeth 42.9	47.3 (43.7 to 50.9)	42.5 (40.9 to 44.1)
Overall QOF achievement (%)	95.7 (92.4 to 97.3)	95.6 (90.5 to 98.5)	94.3 (92.7 to 95.3)
Clinical QOF achievement (%)	95.1 (91.5 to 96.8)	94.4 (89.6 to 98.1)	94.7 (92.4 to 95.7)
Public health QOF achievement (%)	98.5 (93.8 to 100)	99.4 (93.1 to 100)	91.5 (89.8 to 96.6)

Figures are median (interquartile range) except where indicated

*IMD* Index of Multiple Deprivation 2010 score, *QOF* Quality Outcome Framework

Table 2 compares the set-up and experience of the automated and in-practice methods. The in-practice method was quicker to set up than the automated approach and the full control of the randomisation procedure was retained with the study team at King’s. However, the in-practice method was labour intensive and required at least one study team member to visit each practice every month. The records for the trial participants had to be stored on practice systems for the study duration as a result data for 10 practice months were lost for the in-practice method. The automated method took longer to set up due to the development time required to integrate new software code for the randomisation procedure into existing software. Without full control over the procedure we were unable to include block randomisation and instead simple randomisation performed monthly stratified by practice was implemented. We were not able to fully audit and document the randomisation process but the advantage was that the records for trial participants were stored centrally at the offices of the Primary Care Shared Services team and all were successfully retrieved at the end of the trial.

**Table 2:** Comparison of in-practice and automated allocation methods

	In-practice	Automated
Time to start	Sooner (3 months)	Later (7 months)
Randomisation design	In-house	In-house/third party
Randomisation conduct	In-house	Third party
Randomisation record	Full	Partial
Labour intensive	Monthly general practice visits over 18 months	No requirement for practice visits
Outcome data	Extracted at general practice visits	Extracted at general practice visits
Missing data	Present for 10/178 (6%) practice months	0/72 practice months
Trial outcomes	Generally consistent	Generally consistent

Health check uptake was 590/4095 (14.4%) for the Standard Invitation trial arm; 630/3988 (15.8%) for the QBE questionnaire trial arm; and 629/3969 (15.9%) for the QBE questionnaire and Incentive trial arms, respectively. Overall, there were no important or statistically significant differences between trial arms with difference in uptake between the Standard Invitation and QBE questionnaire trial arms found to be 1.4% (95% CI − 0.1 to 3.0%; *P* = 0.070) and Standard Invitation and QBE questionnaire and Incentive arm 1.5% (95% CI − 0.0 to 3.1%; *P* = 0.054). The two intervention arms were found to have similar uptake with an estimated difference − 0.01% (95% CI − 1.59 to 1.58%; *P* = 0.995).

**Fig. 2:**
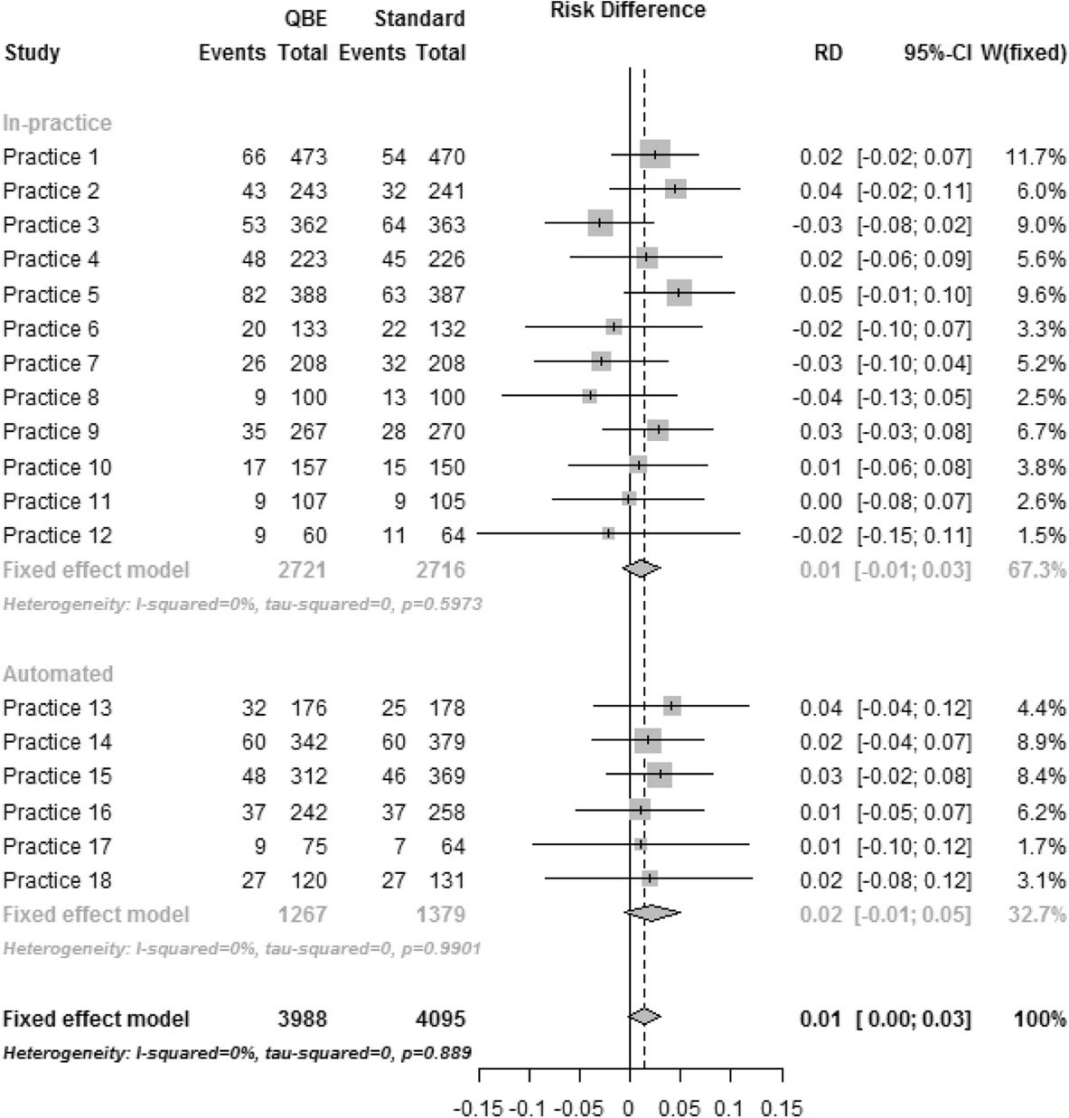
Standard care versus standard care + QBE questionnaire. Forest plot displaying the intervention effect (risk difference) by practice and randomisation method for comparison standard care versus standard care + QBE questionnaire

**Fig. 3:**
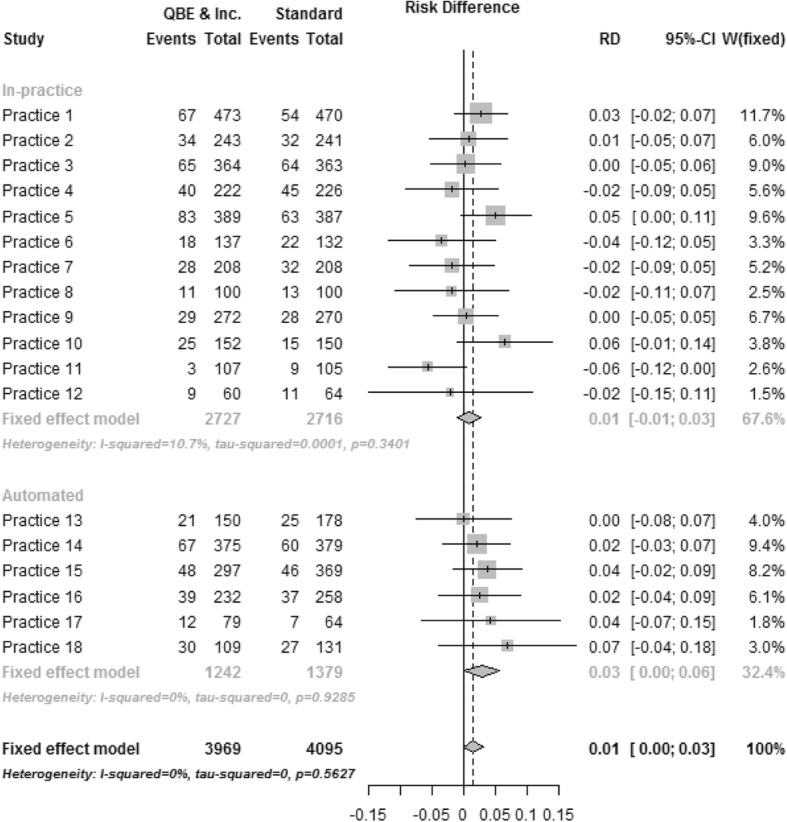
Standard care versus standard care + QBE questionnaire + Incentive. Forest plot displaying the intervention effect (risk difference) by practice and randomisation method for comparison standard care versus standard care + QBE questionnaire + Incentive

The forest plot in Fig. 2 depicts uptake by practice and method of randomisation for the Standard Invitation versus the QBE questionnaire arm. No heterogeneity was detected over all practices or within randomisation method and the I^2^ statistic was estimated to be zero. The difference in uptake between arms for both randomisation methods was similar with an increase of 1.06% (95% CI − 0.08 to 2.94%) for the in-practice method and 2.19% (− 0.59 to 4.96%) for the automated method. Similar results were seen for the other two comparisons. In the Standard Invitation versus the QBE questionnaire and Incentive arm (Fig. 3) increase in uptake by the randomisation method was 0.84% (95% CI − 1.03 to 2.71%) and 2.84% (95% CI 0.03 to 5.67%) in the in-practice and automated methods, respectively, with the I^2^ statistic 10.7% in the in-practice method but zero in the automated method over all practices. In the QBE questionnaire versus the QBE questionnaire and Incentive arm (Figure not shown) the uptake by randomisation method was − 0.21% (95% CI − 2.11 to 1.68%) and 0.68% (95% CI − 2.26 to 3.62%) in the in-practice and automated methods, respectively, with the I^2^ statistic estimated to be zero over all practices and by randomisation method.

## Discussion

This trial was conducted as a rapid trial with participant recruitment, randomisation and outcome assessment being completed using primary care EHR. We have successfully demonstrated the feasibility of utilising an automated recruitment and randomisation procedure for an individually randomised controlled trial in primary care. This was achieved by incorporating it into existing software used to identify and invite participants to the NHS Health Check programme. The integration was achieved by negotiating with the borough teams, the commercial information technology company, the Primary Care Shared Services team and general practices to introduce modifications into the software. A fixed-effects meta-analysis showed no evidence of heterogeneity between estimates of effect for randomisation method suggesting that consistent results were obtained for the two randomisation methods.

Whilst the automated process took an additional 3 months to set up, the in-practice method required 178 person-days in practice visits to 12 practices over an 18-month recruitment period. Consequently, we conclude that the trial could have been completed with lower research costs if the fully automated method had been used for all general practices. An additional advantage of the automated method was the ability to store participant’s records centrally and they were all successfully retrieved at the end of the study whereas the records for the participants in the in-practice method were required to be held locally and resulted in the loss of 10 practice months’ worth of data. However, the lack of full control by the study team for the automated procedure meant that the design of the randomisation was determined in part by the provider, and block randomisation, used in the in-practice method, was not included. The automated procedure was not fully auditable unlike the in-practice method where a full record of the randomisation was retained.

Our experience suggests that there may be both advantages and disadvantages to programmed methods for automated randomisation. It may not always be possible to fully anticipate the consequences of adopting a given procedure. Consequently, it will always be desirable to conduct robust pilot investigations of such procedures to ensure that a full trial can be successfully delivered. Our experience also suggests that active engagement with health service and information service providers and other stakeholders will often be essential. In the present trial, there was no requirement for individual patient consent but more sophisticated approaches to randomisation may be required in future trials in order to ensure that more restrictive ethical and information governance requirements are met.

Investigators who examined their experiences of conducting two point-of-care trials that included automated randomisation and recruitment methods identified a number of challenges relating to the complexities in obtaining research governance approvals [10]. They made several recommendations to simplify the trial recruitment and consent procedures in order to improve future efficacy of such trials. Due to the nature of our intervention the present trial did not require individual participant consent, consent was provided by the partner at the practice and as a result the adaptation to the software was relatively straightforward. Given the potential gains in efficiency made utilising automated randomisation and recruitment into clinical trials there would seemingly be great benefit to implementing the recommendations made by Van Staa et al. [10] to simplify research governance approvals.

## Conclusion

We have demonstrated the feasibility and increase in efficiency of undertaking automated recruitment and randomisation in an individually randomised trial performed in primary care. Similar approaches might now be extended to other contexts and services.

## Additional file

Additional file 1: Three-arm superiority randomised controlled trial design. (DOCX 29 kb)

## Abbreviations

CVD: Cardiovascular disease
GP: General practitioner
HTA: Health Technology Assessment
NHS: National Health Service
NIHR: National Institute for Health Research
PNL: Pre-notification list
QBE: Question-behaviour Effect
