# Supplementary material for: Automated recruitment and randomisation for an efficient randomised controlled trial in primary care
Source: Trials. 2018 Jun 27;19:341. doi: 10.1186/s13063-018-2723-3 (PMC6020316; doi:10.1186/s13063-018-2723-3)
Supplement: Supplementary file 1 — Three-arm superiority randomised controlled trial design. (DOCX 29 kb) [file 13063_2018_2723_MOESM1_ESM.docx]

**Borough 1:** 50 general practices

**Borough 2:** 45 general practices

*Approximately 35,480 participants eligible and invited for an NHS Health Check annually.*

60% of general practices expected to consent to participate in study

12 practices purposely selected to participate in the **in-practice method**

6 practices purposely selective to participate in the **automated method**

Block randomisation stratified by practice performed once monthly

Simple randomisation stratified by practice performed once monthly

**Trial arm A** (33.3%). Standard care

**Trial arm B** (33.3%). QBE questionnaire plus standard care

**Trial arm C** (33.3%). QBE questionnaire, incentive plus standard care

**Trial arm A** (33.3%). Standard care

**Trial arm B** (33.3%). QBE questionnaire plus standard care

**Trial arm C** (33.3%). QBE questionnaire, incentive plus standard care

Seven day interval

Seven day interval

Standard invitation letter and information sheet sent, as standard, by the central unit who organises NHS Health Check invitations

Reminder letter after 12 weeks sent, as standard, by the central unit who organises NHS Health Check invitations

**Outcome evaluation**: difference in uptake of the NHS Health Check at 6 months
